# Loss of the Pericranium and Its Reformation without Necrosis or Exfoliation of the Cranial Bones

**Published:** 1864-12

**Authors:** Le Grand Capers

**Affiliations:** Surgeon P. A. C. S.


ART. II.—*Loss of the Pericranium and its Reformation without Necrosis or Exfoliation of the Cranial Bones.* By LE GRAND CAPERS, Surgeon P. A. C. S.

In support of the views recently advanced by Surgeon E. S. Gaillard, in his article on "The Relations of the Periosteum to Osteogenesis," the following case is submitted.

It is proper to state, as a prefatory remark, that Surgeon E. S. Gaillard, in the article to which allusion is made, claims that "no fallacy is more frequently accepted as truth, than that the periosteum is necessary to osteogenesis: that it is essential for the vitality and reparation of bone." He questions the truth of the dogma so frequently taught in the library and in the lecture-room, that "denuded bone will die," and endeavors to prove, by reasoning and illustration, that denuded bone, on the contrary, will be recovered and its vitality remain, as a rule, uninjured.

This important subject, the economy of life and limb, can not be too frequently brought to the attention of the profession, or the truth advocated be too fully illustrated. It is in this connection that the following case is submitted.

The case was one in which the entire scalp (integuments and pericranium) was forcibly removed, from frontal protuberance, around over the ears and across the superior semicircular ridge of occiput, by that barbarous process termed "scalping."

The bone, thus denuded, was seen and felt by the officers in charge, Assistant-Surgeons A. K. Smith, Vollum and Swift, U. S. A., and by numerous officers of the garrison, as well as by myself. Over this extensive surface, granulations soon formed, sprang up, as it were, and in less than four weeks the entire bones were covered and protected by a thick, fleshy, granulating surface, covering every vestige of bony matter; *this without exfoliation or necrosis.* It would seem still more remarkable from the fact that the patient, a Mexican boy of eighteen years, having been left for dead by the Indians who committed the deed, was alone, sixty miles from the nearest post fort, "Phantom Hill," and was several days in making the journey; the parts, during the interval, being exposed to the sun, night-air and dust of the road. From these causes several days intervened ere he received the slightest attention. Immediately before losing his scalp, the patient received a rifle-ball wound at the lower and posterior part of the neck, which for some time rendered him insensible. The ball fractured the spine of one of the cervical vertebræ, traversed the neck and arm, emerging near the elbow, in its course fracturing the outer half of the clavicle and also the humerus. A point of interest, and one which possibly may prove instructive, is the statement of the patient, that to arrest hemorrage, having nothing else at hand, he applied to his wounds tar and grease from the tar bucket of his wagon Could this mixture have exerted any influence upon the denuded bone in causing granulations, or was it an inflammatory action of the bone itself causing effusion of lymph with subsequent organization? Could this mixture have acted primarily as a styptic? In conclusion, I may mention that the patient died about four months after the injury, from the effects of the wound of his spine. The granulations of the head were completely covered with cuticle by the sixth week, after which he experienced not the slightest inconvenience from the injury to the scalp; the pericranium having been completely restored, and the vitality of the denuded cranial bones not having been in any respect impaired.

It would be advantageous to the profession, for others to contribute their testimony also in regard to this interesting subject.

